# Chicago Medical Society

**Published:** 1882-01

**Authors:** 


### ARTICLE XI.

CHICAGO MEDICAL SOCIETY. Stated Meeting, November 21, 1881. Dr. E. Ingals, President, in the Chair.

### PREDISPOSING CAUSES OF THE ALCOHOL AND OPIUM HABITS.

Dr. Mary H. Thompson read a paper having the above title. She started from the fact that dietary measures are as important in the human economy as surgical treatment. The use of too much wine at the table was in both sexes a frequent cause of intemperance. Another cause was found in the frequent prescribing of alcoholics and opiates to alleviate suffering, especially in women and in children, who form four-fifths of the ordinary patients. After a while, the patient prescribes for herself in hysterical troubles, slight headaches; or takes alcohol or opium simply as a stimulant. Then intoxication is a most frequent cause of accidents. It was a wiser measure to encourage the patient to bear pain until the cause of her disease was removed, and very unwise for mothers to dose their infants with paregoric or other opiates in order to induce sleep. She related a few cases of chronic opium poisoning in infants referable to the above cause.

Whisky is often a substitute for opium. It was an ascertained fact that patients had begun taking cod-liver oil with whisky, but soon dispensed with the oil and kept up the whisky. As a method of alleviating pain, she proposed that of Emmet, which consists in the external application of heat in the form of hot water to the extremities. She thought opiates should not be given in inflammations, nor after surgical operations, nor in septicæmia.

Females were more predisposed to form the opium habit, probably on account of a greater susceptibility to pain.

Hereditary tendencies and various circumstances act also as causes.

The action of narcotics is in ratio of the development of the brain, and their dose is not well ascertained. Dr. Thompson had met many cases of opium habits, and she knew several ladies, some from the best society, who had entered the hospital in order to be fed with opiates. Some accumulated doses until a couple of grains were reached, which they took at one dose, to get the full effect of the drug. The doctor proposed the following questions, which remained unanswered:

1. What was the immediate effect of alcoholics on the liver?
2. How can we prevent the habits?
3. And after these have formed, how should they be remedied?

DISCUSSION.

Dr. Odelia Blinn stated that overwork, which is a characteristic of the people of this country, was a most frequent cause of intemperance. Another cause was the poor diet on which the working class fed; they endeavored to supplement the quality of their food by a large imbibition of alcoholics. Her experience had been that a moderate use of diluted alcohol increased the appetite and helped digestion in healthy people, and was especially useful when the vitality was sinking in various diseases. Narcotics, she thought, were too easily procured by the people at large.

Dr. S. R. Millard agreed with the lecturer, that prescribing narcotics was a frequent cause of the opium habit.

Dr. Allport thought that the external use of heat, as recommended by Dr. Thompson, produced a withdrawal of blood from congested centers, and was a most efficient palliative of pain.

Dr. S. J. Avery said that we had not yet found a substitute for the anodynes. In pneumonia, he had found alcohol the best remedy; and the same was true of opium in peritonitis. Instead of creating an appetite for stimulants, it had been the reverse; his patients had never kept up the habit. He believed in hereditary tendencies.

President E. Ingals, an absolute believer in temperance, thought he had been fortunate not to come across any habitual drunkard or opium eater who could refer his habits to a physician's treatment. He always prescribed opium and alcohol in diseases in which he expected good from their use, except in cases of reformed drunkards.

Dr. C. W. Purdy stated that an infusion of absinthe was a good substitute for alcohol whenever one wished to break the habit of drinking. It should not be taken too long. He believed that opium acted as something specific in inflammation, and that nothing equalled Dover's powders to break a cold.

Dr. W. H. Curtis read a paper, an abstract of which follows:

### DISPOSAL OF THE DEAD.

Cremation among the Greeks and the Romans was reserved to the chiefs and considered a high honor, but it also solved the problem of a quick destruction of the dead during the wars of the empire against the barbarians. The first Christians buried their dead in the catacombs, and the wish to be buried with martyrs and saints gave rise to intra-mural burying. After all, it is a difficult matter to convince the people that death levels all. Burial does not render the body innocuous, but is a cause of pollution of the water, of the air, and a direct danger in opening graves.

The question of interest on the part of societies and trades had always been powerful. Laws prohibiting intra-mural burying grounds were early edicted by popes and bishops. Epidemics of pestilence due to that cause have been referred to by Ambrose Parè. However, the lecturer did not believe that the exhalations from dead bodies could give rise to any one disease in particular, but fed the germs which facilitated the spread of all the infectious and filth diseases. Dr. Curtis then related the experiments of Pasteur in anthrax, and the manner in which the germs of the disease are brought to the surface by earth-worms which feed on cadavers. (See the JOURNAL, November, 1880). He was satisfied that the germ theory explained everything concerning infectious diseases. That the dead kill the living is only too true. The decay of organic matter is proven to be a most common cause of disease and death. How, then, shall we dispose of the dead? It is a false sentiment which is manifested for inhumation. There is no possibility for our bones to be left undisturbed. Millions have been buried in small graveyards. Body snatching is on the increase, and no one is protected against it by inhumation. And the security of the dead necessitates such expenses as cannot be borne by the poor. Besides, in every one exists at certain times some dread of being buried alive In some it becomes a subject of paramount importance. We can seldom reach a positive evidence of death. But cremation would remedy all this. It is the only advisable means for disposing of the dead without any harm to the living.

The disadvantages are few. Cases of poisoning are rare, and the detection of vegetable poisons is practically impossible in the exhumed body. Cremation was costly in the pursuance of rites, but very cheap in the burning of martyrs.

By the Siemens process the body does not come in contact with the fire, but is cremated by a great heat generated outside the retort.

The cost would average fifteen dollars, and would thus be very accessible. However, expenditures were of little account, and people will, as ever, lavish money on the dead. The lecturer considered cremation a present necessity, while it was at the same time a mark of progress. There was no room, and should not be, for such plagues as our present cemeteries. "Man should become a handful of ashes, and nothing more."

In America, there was nothing but individual sentiment and prejudice against cremation. There was no law against it, and the American people are so practical that their notions are readily turning in favor of cremation.

Dr. Roswell Park exhibited a piece of wood $2\frac{1}{2}$x$\frac{1}{2}$ inches in size, which he removed from the superior maxilla and zygomatic fossa of a child fourteen years old. It had been retained fifteen months, and had given rise to sinuses in the soft palate, in the nose, and beneath the orbit.

Meeting held October 3, 1881. Dr. E. Ingals, President, in the Chair.

### IS THERE A CHRONIC CARBOLIC ACID POISONING?

Dr. Andrews read a practical paper on the above question. He began the use of the antiseptic treatment fifteen years ago. A few objections had since arisen as to the use of carbolic acid. The spray was a fine mist of steam, which did not always kill bacteria, which, it has been proven, pass sometimes through it intact, though they always die when they come in contact with the surface of the body wet from a condensation of the carbolized steam; a lotion of carbolic acid, then, is all that is necessary. He had performed but few ovariotomies, but he believed the spray very efficient in such cases. The use of the McIntosh cloth sometimes produced an intense heat, partly from impeding perspiration, and this had proven a serious difficulty whenever a large surface of wounded tissues was exposed, as in amputation of the breasts. In such cases, he removed the dressings more frequently. Thus, many of his patients had their dressings renewed twice a day. But there must have been a greater absorption of carbolic acid under these circumstances. After a week or ten days he has met with vomiting and irritation of the stomach in some cases. Was that due to a sort of chronic carbolic acid poisoning?

Not long ago, a patient had both of her breasts removed for the second time on account of sarcoma. The McIntosh cloth was not used. Vomiting set in the tenth day. The urine was not darker than usual. The doctor replaced carbolic by salicylic acid, and the patient got well.

Another patient presented himself at the hospital with a fluctuating abscess in the thigh, which proved a psoas abscess. Dr. Andrews aspirated. Later, mortification set in, which was treated with injections of carbolic acid, but these could not reach the deeper parts of the abscess. After a week or two, vomiting set in, and continued after salicylic had replaced carbolic acid. Has seen many cases resulting in that same manner. Carbolic acid being an alcohol, may it not produce a sort of intoxication in its systemic effect? Or does it act only as a local agent? It is asserted that carbolic acid resembles arsenic in this last particular. Thymol he found more irritating when used in $2\frac{1}{2}$ per cent. sol., while he does not believe in its efficacy if used one part in five thousand.

Dr. Andrews made the suggestion, as a conclusion, that antiseptic dressings should be left undisturbed for days, as Lister proposed.

Dr Holmes presented an abstract of two cases which have been more fully reported in Knapp's Archives of Ophthalmology.

I. Mrs. H., forty years of age, while sitting quietly with friends, experienced a very sudden excruciating pain in the left orbit, followed immediately by great swelling and protrusion of the globe between the lids. All motion of the eye, as also its vision, was destroyed. A loud aneurismal murmur was at once observed by the patient. This could be distinctly heard when the ear was placed on the head, especially near the left orbit. On account of the shock and continued excessive prostration, surgical interference was delayed from day to day.

By the request of Prof. Etheridge, the attending physician, I had an opportunity of examining the eye two weeks after the attack. At this time the patient seemed too weak for the treatment of aneurism by the use of veratrum viride or by injection of a styptic into the sack. Pain was relieved by opium, which was well tolerated. The cornea, which was becoming opaque, was kept moist and protected from the air. The propriety of ligating the carotid artery was considered in consultation with Prof. Gunn, when, fortunately, in two days, the symptoms began spontaneously to abate. At the end of two months the pain, swelling and pulsating sounds had entirely disappeared. The globe was atrophied.

As the patient began to improve, it was observed that there was almost complete paralysis of the left side of the face. There appeared also, at the same time, loud râles in the left side of the chest, attended by a severe cough. These symptoms continued through life, although the general health became unusually good.

At the end of two years Mrs. H., without the slightest premonition, died suddenly at the breakfast table. No autopsy was obtained. Although no disease of the arterial system could be detected, there was undoubtedly a weak condition and rupture of an artery in the orbit, and finally in the brain.

II. Traumatic loss of the iris.

Mr. D., aged sixty years, received a severe blow on the right eye from an iron lever. There was considerable hæmorrhage, with total loss of sight and great pain. The latter symptom speedily subsided. Vision returned sufficient to enable the patient to walk alone in localities in which he was acquainted.

Two years after the accident, in examining the patient for a disease of the other eye, I learned the short history just recorded. I observed that the iris was entirely absent in the right eye. Near the periphery of the cornea, at the inner angle, was a small dark scar in the conjunctiva, the seat, probably, of the rupture of the walls of the globe, through which the iris was forced.

The lens remained in its normal position, slightly cataractous. Accidents of this nature are very rare.

